# A New Vegetation Index Based on Multitemporal Sentinel-2 Images for Discriminating Heavy Metal Stress Levels in Rice

**DOI:** 10.3390/s18072172

**Published:** 2018-07-06

**Authors:** Zhijiang Zhang, Meiling Liu, Xiangnan Liu, Gaoxiang Zhou

**Affiliations:** School of Information Engineering, China University of Geosciences, Beijing 100083, China; Zhangzhijiang1012@163.com (Z.Z.); liuxncugb@163.com (X.L.); zhougaoxiang403@163.com (G.Z.)

**Keywords:** heavy metal stress, Sentinel-2, red-edge, spectral indices, multitemporal monitoring model

## Abstract

Heavy metal stress in crops is a worldwide problem that requires accurate and timely monitoring. This study aimed to improve the accuracy of monitoring heavy metal stress levels in rice by using multiple Sentinel-2 images. The selected study areas are in Zhuzhou City, Hunan Province, China. Six Sentinel-2 images were acquired in 2017, and heavy metal concentrations in soil were measured. A novel vegetation index called heavy metal stress sensitive index (HMSSI) was proposed. HMSSI is the ratio between two red-edge spectral indices, namely the red-edge chlorophyll index (*CI_red-edge_*) and the plant senescence reflectance index (PSRI). To demonstrate the capability of HMSSI, the performances of *CI_red-edge_* and PSRI in discriminating heavy metal stress levels were compared with that of HMSSI at different growth stages. Random forest (RF) was used to establish a multitemporal monitoring model to detect heavy metal stress levels in rice based on HMSSI at different growth stages. Results show that HMSSI is more sensitive to heavy metal stress than *CI_red-edge_* and PSRI at different growth stages. The performance of a multitemporal monitoring model combining the whole growth stage images was better than any other single growth stage in distinguishing heavy metal stress levels. Therefore, HMSSI can be regarded as an indicator for monitoring heavy metal stress levels with a multitemporal monitoring model.

## 1. Introduction

Over the past decades, with the rapid development of China’s industry and urbanization, the problem of soil heavy metal contamination caused by industrial and domestic wastewater discharge, sewage irrigation, and automobile emissions has increased in severity [1]. Excessive heavy metal concentrations hinder crop growth and pose a serious threat to human health by entering the food chain and migrating into drinking water sources [2]. According to a partial tally of results, more than 12 million tons of grains are contaminated by heavy metals in China every year [3]. Thus, the rapid and accurate detection of heavy metals in crops is vital to diagnose suspected contaminated areas, assess health risks and consequently protect human health [4]. The conventional methods for monitoring heavy metal stress are based on extensive field sampling and laboratory chemical analyses [5]. These processes are time-consuming, expensive, and unable to monitor contamination over large areas [2,3,4].

Recently, remote sensing technology has provided a cheap, rapid, and environmentally friendly alternative for estimating heavy metal stress [4,6]. Numerous studies have proved that remote sensing technology has been used successfully to monitor heavy metal stress levels in plants [6,7,8,9,10,11]. Heavy metal stress can negatively influence the growth of plants; for example, it can reduce canopy chlorophyll content and change cellular structure [12,13,14,15,16]. Thus, chlorophyll content can be considered a bio-indicator of the actual health status of plants [17,18,19]. The changes in the chlorophyll content can alter the reflectance of visible and near-infrared regions. The red-edge region (i.e., the spectral region of rapid change in reflectance of vegetation from the visible red to the near-infrared range of 680–760 nm) is closely related to the chlorophyll content of various plants [10]. The red-edge region has received much attention for many years from scholars who intend to understand the spectral properties of plants under heavy metal stress. In the early 1980s, their laboratory studies led Horler et al. (1983) to recommend the red-edge spectral region as a possible indicator of heavy metal stress. The red-edge position, which is defined as the wavelength of the inflection point in the red-edge region, is important in detecting heavy metal stress [20,21,22].

However, the vegetation spectral information related to chlorophyll content is often confounded by other factors, such as vegetation cover, leaf area index, aboveground biomass, and soil background [4,23,24]. The vegetation index calculated from two or more wavebands may be adopted to overcome these problems and enhance spectral features [25,26]. Numerous studies have indicated that vegetation indices based on red-edge regions have been developed and used to detect vegetation heavy metals stress successfully. Varaprasad et al. (2016) indicated that the combined index transformed chlorophyll absorption reflectance index (TCARI)/optimized soil adjusted vegetation index (OSAVI) exhibited high resistance to LAI and soil backgrounds compared with single red-edge indices and can thus be useful in monitoring arsenic mitigation in contaminated rice fields [27]. Hui et al. (2017) found that the value of the red-edge normalized index (NDVI_705_) was significantly correlated with the levels of heavy metal stress in crops [28]. These findings suggested that the use of red-edge indices could help improve the detection accuracy of heavy metal stress in crops. Other red-edge indices based on chlorophyll content were reported in previous studies but not to monitor heavy metal stress, such as red-edge chlorophyll index (*CI_red-edge_*) [29], plant senescence reflectance index (PSRI) [30], inverted red-edge chlorophyll index [31], and simple ratio pigment index [32]. Nevertheless, these indices were based on ground-based hyperspectral data rather than satellite data. These hyperspectral indices are often limited to field-scale studies as they encounter difficulty in monitoring heavy metal stress on a large scale.

Satellite-derived vegetation indices provide one of the best possible means to obtain the biophysical parameters of vegetation over large areas (regional or global) whilst retaining high temporal coverage. Thus, their development is of considerable importance. However, until recently, only a limited number of hyperspectral satellite platforms provided radiance data in the red-edge portion of the spectrum, and multispectral satellites, such as Landsat, did not provide red-edge information [33]. Red-edge bands have thus far been typically restricted to commercial satellites, such as the RapidEye and DigitalGlobe WorldView-2 satellites [34]. Recent advances in technology have produced innovative remote sensing sensors, such as the Sentinel-2 satellites, creating new opportunities for heavy metal stress monitoring. Sentinel-2 multispectral instrument (MSI) with refined spatial resolution (10 and 20 m) allows for improved and accurate monitoring of heavy metal stress. Furthermore, the presence of three red-edge bands, centered at 705 (band 5), 740 (band 6) and 783 nm (band 7), which are not present in freely available multispectral sensors, widens the spectral windows for heavy metal stress discrimination at broader scales. The sensor is a polar-orbiting one that acquires high-resolution superspectral images at the nadir position, covering a 290 km field of view, at a high temporal resolution of five days [35,36]. Thus, high temporal resolution can provide important advantage in capturing the dynamic information on vegetation state.

In recent years, the use of multitemporal information has proven useful for improving the classification accuracy in agricultural crops and other vegetation types. Therefore, priority should be given to select effective methods to make good use of multitemporal information for detecting different heavy metal stress levels in rice. It is well-known that RF algorithm, an ensemble technique, was widely used in land-cover classification and estimation of biophysical properties [37]. Gomariz-Castillo et al. (2017) concluded that a combination of multiple Landsat images using random forest from four seasons can significantly improve the accuracy than one-season in a highly fragmented semiarid area [38]. Clark (2017) indicated that there were 1.7% to 20.9% significant improvements in overall accuracy with multiseasonal (spring, summer, and fall) over summer-only images for random forest in mapping regional land-cover [39]. Chrysafis et al. (2017) assessed the utility of single-date, single-season (dry, wet), and multitemporal (May–December) images for estimating forest stand parameters in a Mediterranean mixed forest, using the random forest regression algorithm. They concluded that the multitemporal and dry-season models were more accurate than the single-date models [40]. Thus, in this study, RF was adapted to evaluate heavy metal stress in rice based on the multitemporal Sentinel-2 images.

In this study, we aimed to develop a new index that can enhance the accuracy of detecting and distinguishing different heavy metal stress levels. A new vegetation index named heavy metal stress sensitive index (HMSSI) was introduced here. After HMSSI calculation, this study focused on (1) comparing the HMSSI with *CI_red-edge_* and PSRI for monitoring heavy metal stress at different growth stages of rice and (2) constructing a multitemporal monitoring model based on multiple Sentinel-2 images and investigating whether multitemporal monitoring model based on multiple growth stages is better than the single growth stage.

## 2. Materials and Methods

### 2.1. Study Area

**Figure 1:**
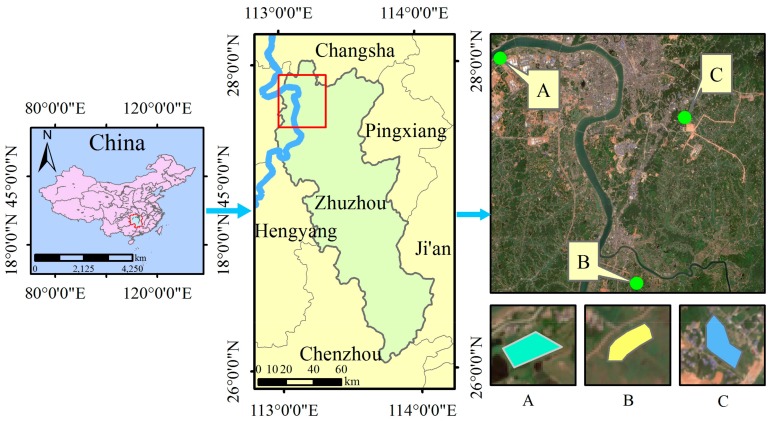
Location of the study areas in the city of Zhuzhou, Hunan Province, China.

The study area for the field experiment is in Zhuzhou City, Hunan Province, China, which is an old industrial base and important grain production region. This area is in a subtropical monsoon climate zone with warm temperatures and sufficient sunlight. The annual average temperature is 16–18 °C and the average precipitation is approximately 1500 mm. The dominant soil texture is red loam with sufficient organic matter (2–3%). Soil organic matter mainly comes from plant, animal, and microbial residues. The dominant crop in this area is rice, which is transplanted in early June and matures in late September. The growth period is approximately 100–120 days. Rice paddies are irrigated mainly by Xiangjiang River. However, the various industries in the region have caused severe heavy metal pollution in the Xiangjiang River. Thus, the rice paddies are contaminated by heavy metals because of irrigation with the river water contaminated by industrial effluent. In this study, three large paddy field areas (labeled A, B, and C) with a size of 1 km × 1 km were selected (Figure 1). In each area, 100 uniformly distributed sample plots were selected in the rice field, and the corresponding positions were obtained using GPS. The area of each sample plot is 10 m × 10 m. Soil heavy metals were collected by soil sampler. The sampling depth was about 20 cm, using bottom-up approach to sample soil. The main heavy metals in this area are cadmium (Cd), lead (Pb) and mercury (Hg). Details regarding the soil heavy metals in this area are shown in Table 1. The concentrations of Cd, Hg, and Pb in the three areas are all higher than the level II soil quality standard values [41], with that of Cd being particularly higher. The concentrations of the soil heavy metals were measured using sampling tests. On the basis of these concentrations the stress levels in areas A, B, and C were determined as ‘high’, ‘medium’, and ‘low’ respectively. The three rice-growing areas exhibit minimal differences in topography and agricultural management; thus, they have similar climates, soil textures, and water. The planted rice in the study areas was supplied with abundant irrigation water and the appropriate amount of fertilizers with uniform distribution to ensure the normal growth of rice, without the impact of other environmental factors.

### 2.2. Sentinel-2 Images

Sentinel-2 programs consists of Sentinel-2A and Sentinel-2B satellites, which were launched on 23 June 2015 and 7 March 2017, respectively [42]. The two satellites all carry a multispectral instrument. The MSI has a swath width of 290 km by applying a total field of view of approximately 20° [43]. With the twin satellites, the revisiting cycle can be shortened to 5 days. However, more than 5 days (i.e., probably several months) are generally required to acquire a cloud-free Sentinel-2 image for specific areas, owing to the cloud and shadow contamination [42]. The Sentinel-2 MSI provides 13 spectral bands in the visible, near-infrared and short-wave infrared wavelengths, with four bands at 10 m (centered at 490, 560, 665, and 842 nm), six bands at 20 m (centered at 705, 740, 783, 865, 1610, and 2190 nm) and three bands at 60 m spatial resolution (centered at 443, 940, and 1375 nm) (Table 2) [44,45,46,47].

In this study, we selected six available cloud-free Sentinel-2 images for the study areas during the growing season of rice in 2017. Amongst them, two images were at the booting stage (12 July and 24 July), two images at the flowering stage (6 August and 21 August), and two images at the mature stage (17 September and 30 September). The Sentinel-2 satellite images (Level-1C) were downloaded from the Copernicus Open Access Hub (https://scihub.copernicus.eu/). These images provided orthorectified top-of-atmosphere reflectance in Universal Transverse Mercator (UTM) projection, with the World Geodetic System (WGS84). The images were resampled into 10 m, which was the highest resolution amongst 10, 20, and 60 m. The atmospheric correction and resampling of the Sentinel-2 images was also performed using the Sen2cor atmospheric correction toolbox, which is a built-in algorithm within the Sentinel Application Platform (SNAP) tool version 5.0. The tool was developed primarily for Sentinel images [48]. The regional spatial distribution of rice paddies was obtained using supervised classification.

### 2.3. Methods

#### 2.3.1. Definition of HMSSI

In this study, two red-edge indices, namely *CI_red-edge_* and PSRI, were selected to establish HMSSI. *CI_red-edge_* was proposed by Gitelson et al. (2003) [29]. The formula of *CI_red-edge_* is as follows:

(1) $$CI_{red-edge}=\left(\frac{R_{783}}{R_{705}}\right)-1,$$

where *R*_783_ and *R*_705_ are the reflectance values in the wavelength of 783 and 705 mm, respectively. For Sentinel-2 imagery, *R*_783_ and *R*_705_ correspond to band 7 and band 5, respectively. The major advantages of *CI_red-edge_* are its linearity with chlorophyll content and the absence of the saturation effect [49]. Low *CI_red-edge_* values indicate low chlorophyll content and severe stress.

PSRI was introduced by Merzlyak et al. (1999) [30]. It can be computed as follows:

(2) $$\mathrm{PSRI}=\frac{\left(R_{680}-R_{500}\right)}{R_{750}},$$

where *R*_680_, *R*_500_, and *R*_750_ correspond to Sentinel-2 band 4, band 2, and band 6 respectively. PSRI is designed to maximize the sensitivity of the index to the ratio of bulk carotenoids (e.g., alpha-carotene and beta-carotene) to chlorophyll. An increase in PSRI indicates increased canopy stress (carotenoid pigment). The values of this index range from −1 to 1, with the common values for green vegetation ranging between −0.1 and 0.2 [50].

Based on these two red-edge indices, the new vegetation index HMSSI was established in this study. It can be calculated as follows:

(3) $$\mathrm{HMSSI}=\frac{CI_{red-edge}}{\mathrm{PSRI}},$$

where *CI_red-edge_* is the value of red-edge chlorophyll index and PSRI is the value of plant senescence reflectance index. As stress levels increased, the value of *CI_red-edge_* decreased, whereas the value of PSRI increased. Therefore, HMSSI, which combines both, enhances the difference in heavy metal stress.

#### 2.3.2. Construction of the Multitemporal Monitoring Model

In this study, we selected RF algorithm to construct multitemporal monitoring model to discriminate heavy metal stress levels. Random forest is an ensemble of learning algorithms proposed by Breiman [51] that is built to handle high data dimensionality effectively and has demonstrated to be an improvement over traditional decision trees [52]. It consists of a set of independent, unpruned decision trees. The RF ensemble uses a bootstrap sample, i.e., 2/3 of the original dataset (referred to as the “in-bag” sample), to train decision trees. The remaining 1/3 of the data is used to compute an internal measure of accuracy (referred to as the “out-of-bag” or OOB error) [39]. To produce the forest of decision trees, two parameters need to be set: The number of unpruned trees to grow, known as ntree; and the number of predictor variables selected, known as mtry [53]. Mtry variables are tested at each node to specify the best split when growing trees. These randomly selected variables produce low correlated trees that prevent over-fitting. In a classification framework, the final classification results are determined by averaging the results of all the decision trees produced. A total of 500 trees were grown each time, and the square root of the number of total input features were used as the number of split variables in this paper.

The classification accuracies of the model were assessed quantitatively by using the confusion matrix, which is a common used method in remote sensing. This study used overall accuracy, which is computed by dividing correctly classified pixels by the total number of pixels, and the kappa coefficient, which considers the whole confusion matrix instead of using only diagonal elements. In addition, producer’s accuracy and user’s accuracy were used to assess the accuracies of individual classes.

## 3. Results

### 3.1. Comparison of HMSSI with CI_red-edge_ and PSRI

**Figure 2:**
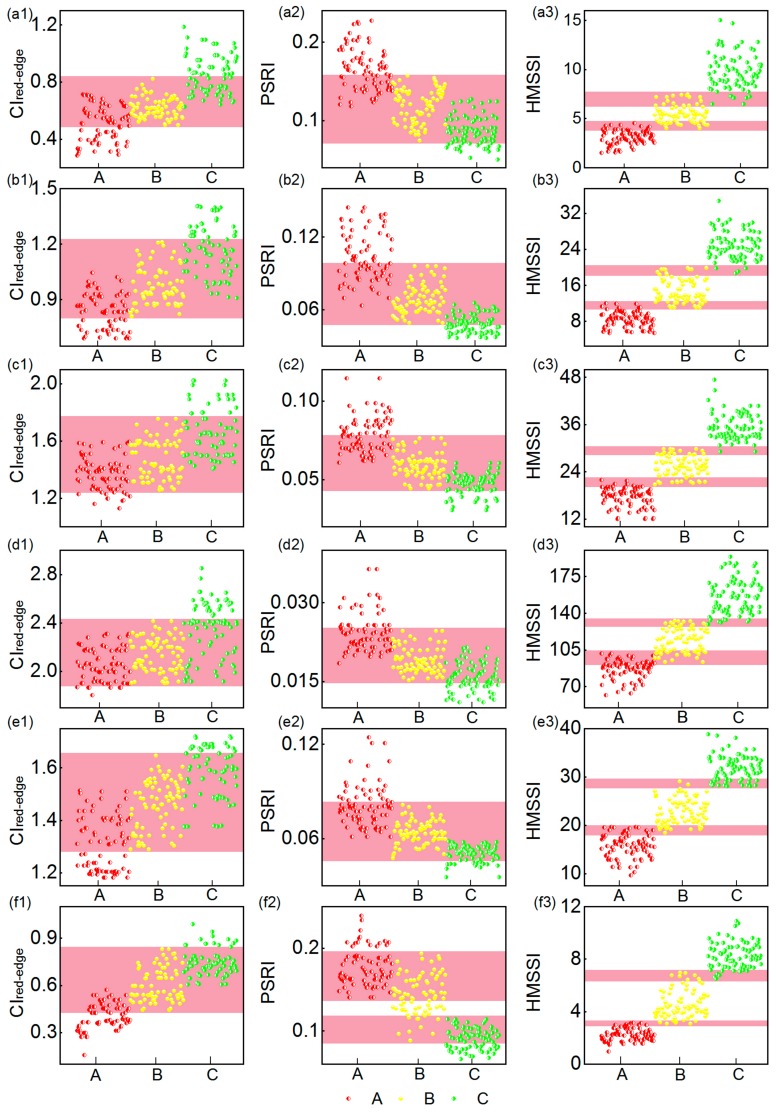
Rice under different stress levels obtained using the three indices at different growth stages. (**a1**–**f1**) The distributions of *CI_red-edge_* under different stress levels at different growth stages; (**a2**–**f2**) the distributions of PSRI under different stress levels at different growth stages; (**a3**–**f3**) the distributions of HMSSI under different stress levels at different growth stages. Note: the pink zone represent the overlapping portion of different stress levels.

To assess the performances of the *CI_red-edge_*, PSRI and HMSSI, 100 rice pixels in every study area were selected. Using Equations (1)–(3), we calculated the three indices of rice under three different heavy metal stress levels at the different growth stages. To depict the differences of HMSSI with *CI_red-edge_* and PSRI in distinguishing the stress levels of rice, their data distributions under different heavy metal stress levels are displayed in Figure 2. Evidently, at the different growth stages, *CI_red-edge_* and PSRI both presented larger overlap regions than HMSSI did in distinguishing different stress levels. The superiority of HMSSI was further assessed by calculating the misjudgment rates of *CI_red-edge_*, PSRI and HMSSI (Table 3). The misjudgment rate is the ratio of the overlapping pixels and sampling points pixels under same stress level. Table 3 suggests that in distinguishing different stress levels, *CI_red-edge_* and PSRI had high misjudgment rates, which were approximate to or more than 60% whereas those of HMSSI were approximate to or below 20%. In particular, *CI_red-edge_* and PSRI nearly failed to distinguish the medium stress levels at the different growth stages.

Through the given analysis, we concluded that neither *CI_red-edge_* nor PSRI can accurately distinguish different stress levels. Apparently, HMSSI exhibited a better distinguishing ability than *CI_red-edge_* and PSRI did. However, HMSSI had a relative high misjudgment rate at several growth stages. Therefore, instead of using only a single stage, multiple growth stages should be combined to detect heavy metal stress.

### 3.2. Performance of the Multitemporal Monitoring Model

In this section, to investigate whether the use of multitemporal images improve the accuracy in discriminating heavy metal stress, we assessed the performance of the multitemporal monitoring model based on Sentinel-2 images of HMSSI at booting stage, flowering stage, mature stage, and the whole growing season, respectively. Thus, the model was employed using four different variable datasets for (1) two images of HMSSI at booting stage; (2) two images of HMSSI at flowering stage; (3) two images of HMSSI at mature stage; (4) six images of HMSSI at whole growth period.

**Figure 3:**
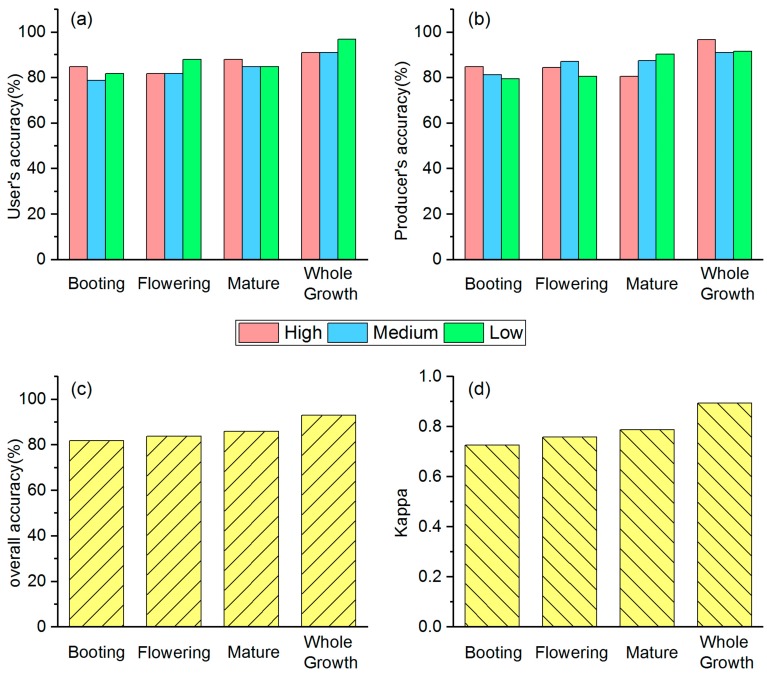
Classification accuracies of multitemporal monitoring model for discriminating different stress levels at different growth stages. (**a**) User’s accuracy of multitemporal monitoring model; (**b**) producer’s accuracy of multitemporal monitoring model; (**c**) overall accuracy of multitemporal monitoring model; (**d**) kappa coefficient of multitemporal monitoring model.

**Figure 4:**
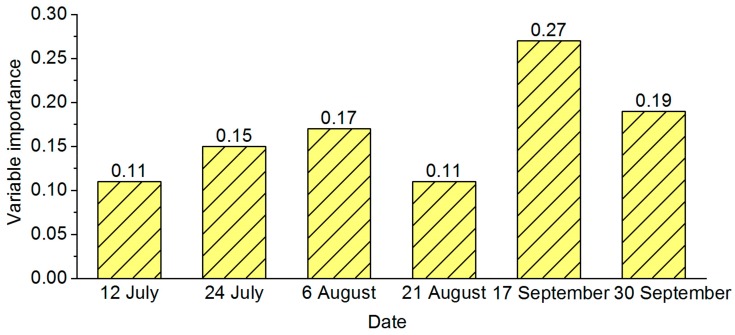
Variable importance of using multitemporal monitoring model based on whole growth stage.

The classification results for multitemporal monitoring model are shown in Figure 3, user’s accuracy of three different stress levels for the whole growth period were 90–96%, whereas for single stage were all below 90%. Similarly, producer’s accuracy followed the same case as user’s accuracy. The highest overall accuracy and kappa coefficient were 92.93% and 0.894%, produced by the whole growth stage of HMSSI. Thus, the whole growth stage combined six images produced the highest accuracy, followed by mature stage, then flowering stage, and finally booting stage. Additionally, for the whole growth period, the importance of each image was shown in Figure 4. Images at different stages had different influences on the model. Obviously, two images at the mature stage took a dominant position, followed by the flowering stage, and then the booting stage.

In summary, the results indicated that the multitemporal monitoring model of HMSSI that combining multiple growth stages had higher accuracy in discriminating the stress levels than the HMSSI with a single growth stage.

### 3.3. Regional Evaluation of Heavy Metal Stress Using HMSSI and Multitemporal Monitoring Model

**Figure 5:**
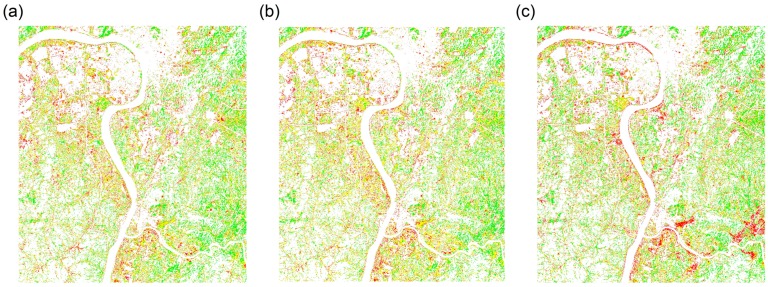

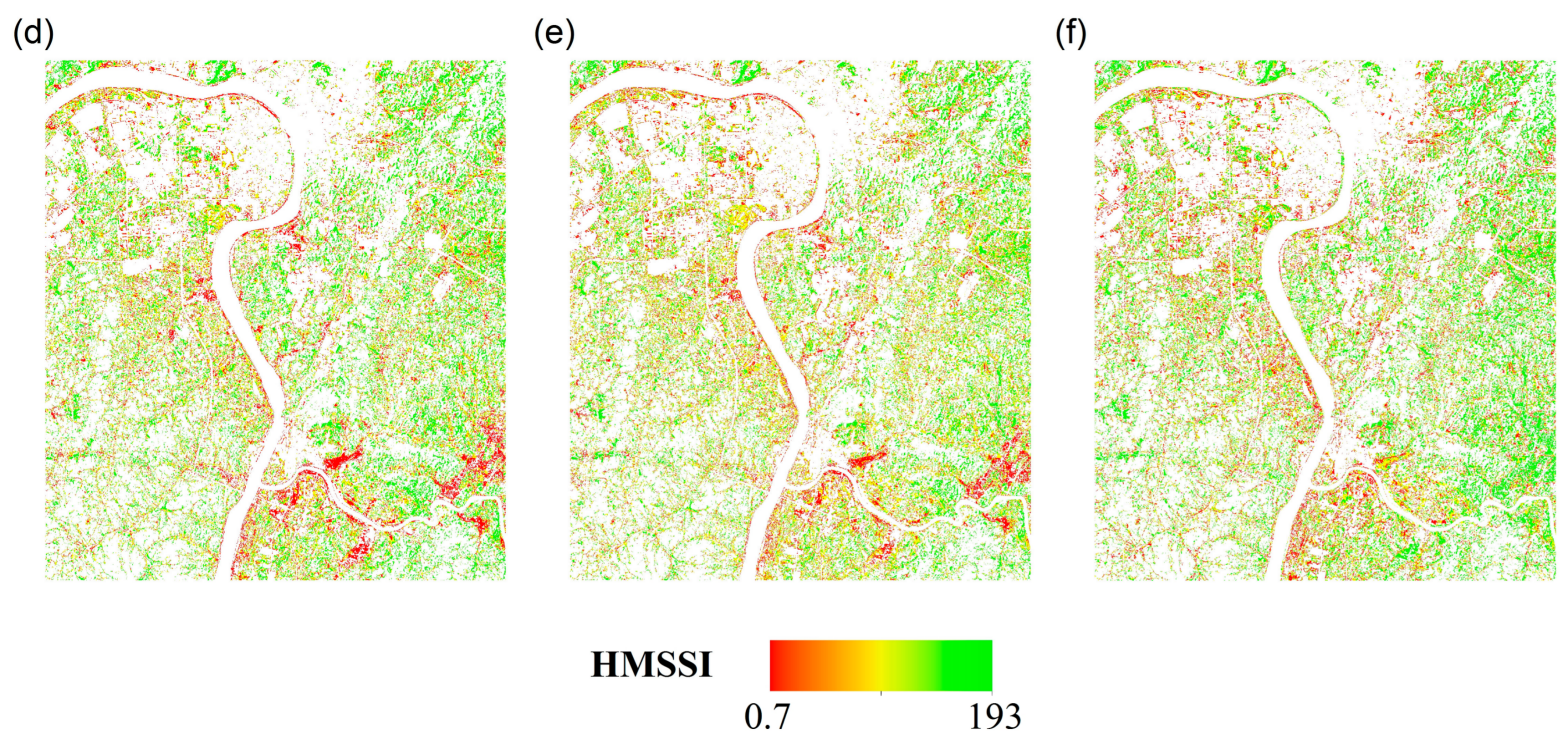
Spatial distributions of HMSSI at different growth stages. (**a**,**b**) Booting stage; (**c**,**d**) flowering stage; (**e**,**f**) mature stage.

**Figure 6:**
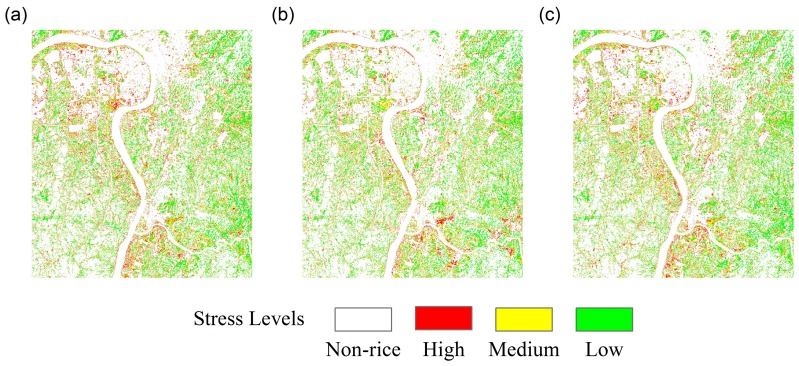
Spatial distributions of stress levels using the multitemporal monitoring model. (**a**) Booting stage; (**b**) flowering stage; (**c**) mature stage.

**Figure 7:**
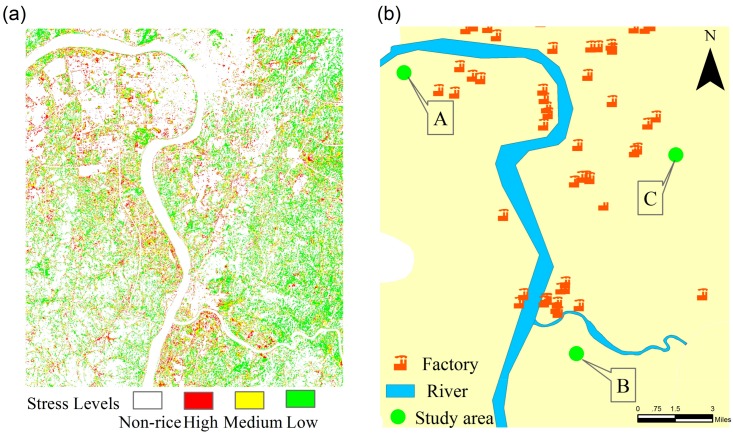
(**a**) Spatial distributions of stress levels using the multitemporal monitoring model at whole growth stage; (**b**) Spatial distributions of factories in the region.

On the basis of the classification results, Figure 5 shows the regional spatial distributions of HMSSI at different growth stages of rice. Figure 6 and Figure 7a show the regional spatial distributions of stress levels on rice using multitemporal monitoring model based on single growth stage and the entire growth period, respectively. Overall, the values of HMSSI in the areas along the Xiangjiang River were lower than in the areas farther away from the river. Moreover, rice subject to high and medium stress levels occurred in the northwest and southeast regions, and near the Xiangjiang River. Meanwhile, the low stress levels were scattered in most of the area in the spatial distribution map, especially in the middle part of the study regions. Figure 7b demonstrates the spatial distribution of factories in this region. Obviously, there are several factories around the study areas. It is noteworthy that study area C is subject to mild pollution for it is located in the high-tech economic zone, factories around it are strictly controlled to discharge pollutants.

Although the stress levels show similar spatial distributions using multitemporal monitoring model based on different growth stage, there are small differences in the area percentage of stress levels. Detailed statistics can be obtained from Table 4. In summary, nearly half of the rice paddies exhibited a low stress level, approximately 30% exhibited a medium stress level, and approximately 20% (lowest) exhibited a high stress level.

## 4. Discussion

The main purpose of this study was to establish a new vegetation index that can enhance the accuracy of detecting heavy metal stress by using several red-edge spectral indices. For that purpose, the data of Sentinel-2 satellites were used. The results of the proposed index were compared with those of existing indices, namely *CI_red-edge_* and PSRI. Notably, the new index named HMSSI can discriminate different stress levels more clearly than *CI_red-edge_* and PSRI can. In addition, based on the new index and multitemporal Sentinel-2 satellite images, a multitemporal monitoring model was constructed to distinguish heavy metal stress levels. The model combining multiple growth stages of rice had higher discrimination rates than the model with only a single growth stage. HMSSI and the multitemporal monitoring model can also be applied to a region larger than the field scale. Therefore, the new index and the model have strong regional applicability.

The satisfactory results can be attributed to two aspects. Firstly, the use of only a single index, such as *CI_red-edge_* or PSRI, which showed unsatisfactory performance in this study, is inadequate to detect heavy metal stress accurately. The values of these vegetation indices exhibited no significant difference under different stress levels on the satellite images. However, these two vegetation indices show opposite trends under different stress levels; *CI_red-edge_* decreases with stress levels, whereas PSRI increases. Being the ratio of *CI_red-edge_* and PSRI, HMSSI can considerably enhance the difference in stress. Secondly, the multitemporal monitoring model based on multitemporal images captures more specific temporal characteristics than the single temporal images; consequently, the established model can accurately classify categories and reduce judgment error. The model performed best with the combinations of multiple images at whole growth stages of rice. This result proves that the multitemporal monitoring model can be applied to monitor heavy metal stress during the growth stage of rice.

Many of the previous studies employed red-edge information based only on narrow-band ground spectral data (≤10 nm), and relatively little is known if broadband red-edge satellite data (>10 nm) can respond to and assist in monitoring heavy metal stress. In this study, we validated the ability of the Sentinel-2 MSI images in discriminating heavy metal stress levels. Several satellite sensors are now equipped with red-edge bands, and the Sentinel-2 satellites are among them. Sentinel-2 provides freely available images. Sentinel-2 is noted for having more spectral bands, the unique red-edge bands (centered at 705 and 740 nm) and refined spatial resolution. The spatial resolution of Sentinel-2 contributes to the precise extraction of rice information. Moreover, the relatively short revisiting cycle of Sentinel-2 means it can obtain more images during the rice growth stage than the Landsat series satellites can. Thus, multiple Sentinel-2 images facilitates the construction of a multitemporal monitoring model.

HMSSI exhibited considerably improved heavy metal stress detection as it combines two red-edge indices. However, this study was conducted under the condition that the stress factor is known beforehand. The mechanism of growth irregularities in crops under external environmental stress is complicated. Many other environmental stress factors (e.g., soil properties, nutrient stress, and water stress) may induce growth irregularities similar to those induced by heavy metals. Until now, discerning heavy metal stress from numerous environmental factors solely with related remote sensing technology and without knowing prior information has been proven difficult [14]. Particularly, a distinct feature of heavy metal stress is that it is generally persistent, whereas other stress factors usually only last for a specific period of time.

In the future, long time series of Sentinel-2 images may be necessary to discriminate heavy metal-induced crop stress from other stressors. Furthermore, future research on the combination of SAR (i.e., Sentinel-1 satellites) and thermal images, which are sensitive to crop structure and physiological function respectively, may help monitor heavy metal stress.

## 5. Conclusions

The advantages of using Sentinel-2 images in evaluating heavy metal stress were investigated. In this study, we determined whether the new proposed index HMSSI can significantly distinguish heavy metal stress levels. The principal results and conclusions obtained can be summarized as follows:

Across stages (i.e., booting stage, flowering stage, and mature stage), the newly developed index HMSSI showed excellent performance compared with the widely used red-edge indices *CI_red-edge_* and PSRI. Therefore, the proposed index can be considered a potential indicator for monitoring heavy metal stress.

A multitemporal monitoring model based on HMSSI was established, and the results showed that the model has a high level of discriminant accuracy for distinguishing stress levels of rice under heavy metal stress.

The new index and multitemporal monitoring model from multitemporal Sentinel-2 images presented strong applicability to detect heavy metal stress in rice on a regional scale.

In conclusion, the new proposed index HMSSI based on Sentinel-2 satellite images provide convenient and rapid chances for practitioners and researchers in detecting heavy metal stress in rice. The multitemporal monitoring model, monitoring consecutively and dynamically, provide the basis for researchers to establish heavy metal stress levels assessment system for a long time.

## Figures and Tables

**Table 1:** Heavy metal concentration (unit: mg kg^−1^) in the soil of the three study areas.

Study Areas	Cd	Hg	Pb	As	Pollution Level
A (27°49′ N, 113°04′ E)	3.54	0.81	120.75	21.35	High
B (27°40′ N, 113°09′ E)	2.31	0.24	91.05	17.34	Medium
C (27°47′ N, 113°10′ E)	0.84	0.35	78.33	10.23	Low
Level II Soil quality standard *	0.3	0.5	300	25	

Note: * Soil quality standard according to the Environment Monitoring Centre of China.

**Table 2:** Spectral and spatial resolution of the Sentinel-2 MSI bands.

Sentinel-2 MSI Bands	Spatial Resolution (m)	Central Wavelength (nm)	Band Width (nm)
Band 1: Coastal Aerosol	60	443	20
Band 2: Blue	10	490	65
Band 3: Green	10	560	35
Band 4: Red	10	665	30
Band 5: Red-edge 1	20	705	15
Band 6: Red-edge 2	20	740	15
Band 7: Red-edge 3	20	783	20
Band 8: NIR	10	842	115
Band 8A: NIR narrow	20	865	20
Band 9: Water Vapor	60	945	20
Band 10: SWIR Cirrus	60	1375	30
Band 11: SWIR	20	1610	90
Band 12: SWIR	20	2190	180

**Table 3:** Misjudgement rates of the three indices at different growth stages.

Vegetation Indices	Growth Stage	High	Medium	Low
*CI_red-edge_*	Booting	64%	100%	48%
		68%	100%	64%
	Flowering	92%	100%	78%
		99%	100%	72%
	Mature	46%	100%	78%
		40%	88%	86%
PSRI	Booting	50%	100%	74%
		52%	100%	50%
	Flowering	54%	100%	78%
		72%	100%	62%
	Mature	70%	100%	80%
		80%	72%	65%
HMSSI	Booting	17%	35%	8%
		8%	32%	5%
	Flowering	6%	28%	3%
		24%	34%	10%
	Mature	18%	18%	26%
		8%	15%	8%

**Table 4:** Area percentage of rice under different heavy metal stress levels at different growth stage.

Growth Stage	Stress Levels		
	High	Medium	Low
Booting	21.76%	27.65%	50.59%
Flowering	21.83%	27.93%	50.24%
Mature	21.65%	28.17%	50.18%
Whole Growth	20.68%	28.45%	50.87%
